# Synthesis, antiinflammatory activity, and molecular docking studies of bisphosphonic esters as potential MMP-8 and MMP-9 inhibitors

**DOI:** 10.3762/bjoc.16.108

**Published:** 2020-06-08

**Authors:** Abimelek Cortes-Pacheco, María Adelina Jiménez-Arellanes, Francisco José Palacios-Can, José Antonio Valcarcel-Gamiño, Rodrigo Said Razo-Hernández, María del Carmen Juárez-Vázquez, Adolfo López-Torres, Oscar Abelardo Ramírez-Marroquín

**Affiliations:** 1 Instituto de Química Aplicada, Universidad del Papaloapan. Tuxtepec, 68301, Mexico; 2 Unidad de Investigación Médica (UIM) en Farmacología, UMAE Hospital de Especialidades, Centro Médico Nacional Siglo XXI (CMN-SXXI), Instituto Mexicano del Seguro Social (IMSS). Av. Cuauhtémoc 330, Col. Doctores 06720, Ciudad de México (CdMx), Mexico; 3 Centro de Investigación en Dinámica Celular, Universidad Autónoma del Estado de Morelos Avenida Universidad 1001, Chamilpa, 62210 Cuernavaca, Morelos, Mexico

**Keywords:** inflammation, molecular docking, organophosphorus compounds

## Abstract

Bisphosphonic acids (or bisphosphonates) have been successfully used in the clinic treatment of bone diseases for over decades. Additionally, the antiinflammatory activity of these compounds has been gaining attention. In our previous work, we synthesized and in vivo evaluated the bisphosphonic esters **1** and **2**, finding a moderate edema inhibition upon oral and topical administration on BALB/c mice. Thus, in this work, the bioisosteric replacement of an amide functional group for an ester afforded the new bisphosphonates **3**–**6**, which had a moderate oral edema inhibition (25 mg/kg dose) and a significant topical antiinflammatory activity (2 mg/ear) on BALB/c mice, with **6** being the most active hit (55.9% edema inhibition), comparable to the positive control (55.5% edema inhibition) on a TPA topical model. Next, to assess the acute toxicity of the synthesized derivatives, test animals were administered with 50–100 mg/kg of **3**–**6**, respectively, by an oral route, and after 14 days, neither lethality nor a significative weight loss were observed. Finally, a structure–activity relationship (SAR) and a molecular docking analysis of **3**–**6** helped us to explain the trend observed in biological tests. Considering all these aspects, we propose the inhibition of MMP-8 and MMP-9 as a possible action mechanism of the synthesized derivatives.

## Introduction

Bisphosphonic acids (or bisphosphonates) are organophosphorus compounds characterized by a P–C–P moiety. These organic compounds are valuable drugs for the treatment of bone diseases as osteoporosis, Paget’s disease, and malignant hypercalcemia [1–3]. Specifically, bisphosphonates act as osteoclast resorption inhibitors, augmenting the bone density and preventing osteoporosis [4]. Moreover, some bisphosphonates have gained attention as potential antiinflammatory agents by in vitro and in vivo assays [5–8]. Additionally, bisphosphonates have been reported as inhibition and downregulation matrix metalloproteinase (MMP) agents [9–11]. In this regard, MMPs are a family of extracellular proteinases (24 isoenzymes in human) involved in tissue regeneration and are closely related to physiologic and physiopathological processes, such as inflammation, angiogenesis, and metastasis in cancer [12–15], pointing at bisphosphonates as potential treatments for cancer and other inflammation-related diseases. In this respect, MMP inhibition by phosphonates or bisphosphonates has been previously studied through computational or X-ray diffraction analyses to describe the enzyme inhibitor site binding modes [16–18].

Thus, in this work, the bisphosphonates **3**–**6** were synthesized by a two-step method and then evaluated through two in vivo acute inflammation models in BALB/c mice. Furthermore, the acute toxicity was determined for these derivatives, and molecular docking studies were performed to account for a possible action mechanism as MMP-8 and MMP-9 inhibitors.

## Results and Discussion

### Chemistry

**Figure 1:**
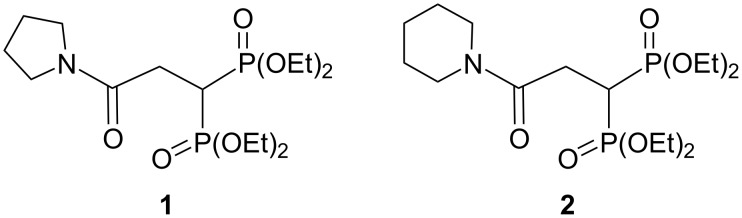
Previously reported antiinflammatory bisphosphonates **1** and **2**. edema inhibition (in %, carrageenan model, 50 mg/kg) for **1**: 7.0; for **2**: 22.2.

As part of our ongoing interest in the discovery of new antiinflammatory agents, our research group have previously addressed the synthesis and in vivo antiinflammatory activity evaluation of the bisphosphonic esters **1** and **2**, observing activity by oral (carrageenan model) and topical administration (TPA model) in BALB/c mice (Figure 1) [19].

**Figure 2:**
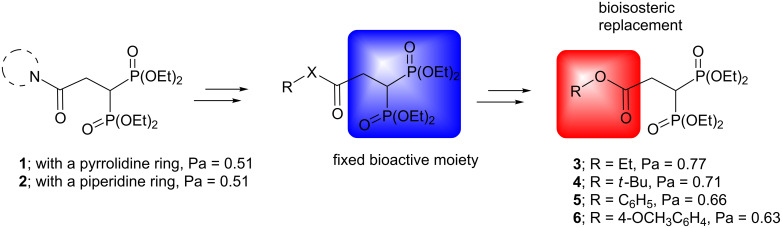
Designed bisphosphonic esters as antiinflammatory agents.

Furthermore, in the search of more potent and low-toxicity derivatives, in this work, we have focussed our attention on the molecular modification of the derivatives **1** and **2** through the bioisosteric replacement [20] of the amide functional group by an aliphatic or aromatic ester. The potential antiinflammatory activity of the new bisphosphonates was predicted using the Prediction of Activity Spectra for Substances (PASSOnline) database, which compares the molecular structure of test compounds vs a large training set of experimental bioactive or inactive compounds [21]. The results of the prediction are summarized as probability of activity (Pa) and probability of inactivity (Pi) values, both ranging from 0 to 1 (Figure 2), where a higher Pa value is desired. Thus, a Pa value of new bisphosphonates ≥ the Pa value of **1** and **2** was the applied inclusion criterion in this study. As can be seen in Figure 2, the Pa is greater for the new derivatives **3**–**6** (0.63–0.77) compared to the previous bioactive compounds **1** and **2** (0.51). In order to explore the SAR in the proposed compounds **3**–**6**, we evaluated the effect of the volume of the ester group by the inclusion of ethyl or *tert*-butyl substituents in the aliphatic derivatives **3** and **4**. On the other hand, the replacement of the aliphatic chains by an aromatic portion led us to the derivatives **5** and **6** where the effect of benzyl or 4-methoxybenzyl substituents was assessed (Figure 2).

**Scheme 1:**
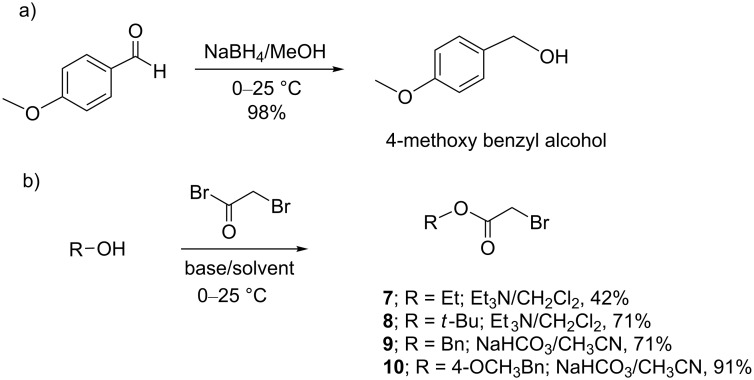
Synthesis of the intermediate bromoaceto esters **7**–**10**.

Furthermore, the bisphosphonic esters **3–6** passed the Lipinski’s rules [22] as criteria for drugs for an oral administration as we wanted to test these derivatives through in vivo acute inflammation models [23]. Then, the above-named derivatives were synthesized in a first stage by the esterification of bromoacetyl bromide and the corresponding alcohol. The reaction of ethyl or *tert*-butyl alcohol and bromoacetyl bromide in the presence of triethylamine in CH_2_Cl_2_ yielded the bromoaceto esters **7** and **8** in 42 and 71% yield (Scheme 1b, method A). Nonetheless, when benzyl or 4-methoxybenzyl alcohol were used under the same reaction conditions, **9** and **10** were obtained in poor yields. Thus, the subsequent use of NaHCO_3_ as a base in CH_3_CN [24] afforded the bromoaceto esters **9** and **10** in 71 and 91% yield (Scheme 1b, method B). It is important to note that 4-methoxybenzyl alcohol was prepared by the reduction of 4-methoxybenzaldehyde (Scheme 1a).

**Scheme 2:**
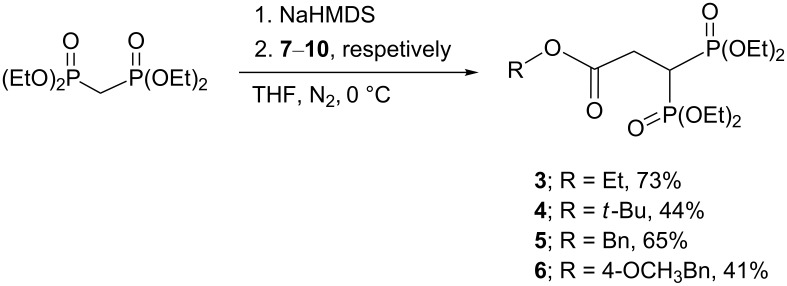
Synthesis of the bisphosphonates **3–6**.

Next, the treatment of tetraethyl methylenediphosphonate with NaHMDS under an anhydrous atmosphere, followed by the addition of **7**–**10**, respectively, afforded the final products **3–6**, respectively, in 41–73% yield (Scheme 2).

### Pharmacological activity

With the target compounds on hand, we proceed to evaluate them with two acute inflammation models: a) 12-*O*-tetradecanoylphorbol-13-acetate (TPA) through topical administration [25] and b) a carrageenan model, through oral administration [25]. When the bisphosphonates **3**–**6** (2 mg/ear) were assayed with a TPA model, the derivatives **5** (40.7% edema inhibition) and **6** (55.9% edema inhibition) were the most active ones, with **6** having a comparable edema inhibition to the positive control (indomethacin, 55.5% edema inhibition, Table 1). Nevertheless, the derivatives **3** and **4** exhibited a moderate antiinflammatory activity (25.5% and 23.9% edema inhibition, respectively). Thus, the inclusion of an aromatic ring in the derivatives **5** and **6** clearly potentiated the desired pharmacological effect on mice. The rationale behind this could be the difference in lipophilicity between the aliphatic esters **3** and **4**, which were less lipophilic (by means of the *c*log*P* value of 0.58 and 1.39, respectively) compared to the aromatic derivatives **5** and **6** (*c*log*P* = 1.80 and 1.85, respectively), with the last being the most permeable one through mice skin (Table 1). Nevertheless, it is important to note that the aliphatic derivatives **3** and **4** considerably differed in the *c*log*P* value between each other (0.58 and 1.39) but had a comparable edema inhibition, indicating that for this study, the volume of the ester group had little importance for the pharmacological activity. More important was the replacement of the aliphatic for an aromatic residue in the ester group, leading to the more active derivatives **5** and **6**. The *c*log*P* value of **5** and **6** was more similar between them, but a remarked difference in the edema inhibition was observed (40.7 vs 55.9%), indicating that the introduction of an electron-donating 4-methoxy substituent on the phenyl ring of **6** potentiated the antiinflammatory activity compared to the nonsubstituted derivative **5**. Next, the DE_50_ value was assessed for the more interesting targets **5** (DE_50_ = 1.4 mg/ear) and **6** (DE_50_ = 0.9 mg/ear), with the methoxy derivative **6** having a higher potency and efficacy (55.9% edema inhibition) in the series (Table 1). Our results were in good accordance with the previous observation of the antiinflammatory activity of the few bisphosphonic esters [26–30].

**Table 1:** Antiinflammatory activity of **3–6** using a TPA topical model. 2 mg/ear of the test compounds was used.^a^

treatment	auricular edema (mg)	% inhibition	*c*log*P*^b^	DE_50_ (mg/ear)

TPA	8.80 mg ± 0.46	–	–	–
indomethacin (2 mg/ear)	3.92 mg ± 0.37^c^	55.5	–	–
**3**	6.56 mg ± 0.19^c,d^	25.5	0.58	n.d.
**4**	6.70 mg ± 0.39^c,d^	23.9	1.39	n.d.
**5**	5.22 mg ± 0.37^c,d^	40.7	1.80	1.4
**6**	3.88 mg ± 0.21^c^	55.9	1.85	0.9

^a^The data is presented as mean ± standard error (s.e.). The percentage of inhibition of the edema is given in respect to the TPA group. Statistical analysis one-way ANOVA, post hoc SNK test (*p* ≤ 0.05). ^b^Calculated using the Molinspiration property engine v2018.10b [31]. ^c^Vs TPA control. ^d^Vs indomethacin; *n* = 5.

Next, the antiinflammatory activity of the bisphosphonates **3**–**6** was assayed with a carrageenan model by intragastric administration. As can be seen in Table 2, the derivatives **3** and **4** were the more active ones this time (24.6% and 20.9% edema inhibition, respectively). A remarkable difference was observed for the derivatives **5** (13.8% edema inhibition) and **6** (9.1% edema inhibition) where a low antiinflammatory activity was observed. In this regard, a clear correlation between the experimental and predicted activity was observed. Thus, the compounds **3** and **4** were predicted to be more bioactive than **5** and **6** (Table 2). Additionally, this tendency was strongly connected to the *c*log*P* value, where the edema inhibition was inversely proportional to the *c*log*P* value (Table 2). Thus, the higher the predicted Pa and the lower the *c*log*P*, the higher the observed activity. It is worth to mention that the oral efficacy of the tested compounds was opposed to that observed with the TPA topical model. This may be a consequence of the lower lipophilicity of **3** (*c*log*P* = 0.58) and **4** (*c*log*P* = 1.39) compared to **5** (*c*log*P* = 1.80) and **6** (*c*log*P* = 1.85), influencing the better dissolution of **3** and **4** in an aqueous medium prior to its absorption through gut mice (Table 2). Finally, the synthesized bisphosphonates **3** and **4** have proven to be more active (24.6% and 20.9% edema inhibition, respectively, at a 25 mg/kg dose) by oral administration than the parent compounds **1** and **2** (7.0% and 22.2% edema inhibition, respectively, at a 50 mg/kg dose, Figure 1) [19]. In addition, **3** and **4** had a comparable activity than what was reported for other bisphosphonic esters [30].

**Table 2:** Antiinflammatory activity of **3**–**6** with a carrageenan oral model. Test compounds: 25 mg/kg.^a^

treatment	paw edema (mm)^b^	% inhibition	*P*a	*c*log*P*^c^

carrageenan	0.830 ± 0.017^d^	–	–	–
indomethacin (20 mg/kg)	0.530 ± 0.04^d^	36.1	–	–
**3**	0.626 ± 0.03^d,e^	24.6	0.77	0.58
**4**	0.657 ± 0.02^d,e^	20.9	0.71	1.39
**5**	0.716 ± 0.03^d,e^	13.8	0.66	1.80
**6**	0.755 ± 0.06^e^	9.1	0.63	1.85

^a^The data is presented as mean ± standard error (s.e.). The percentage of inhibition of the edema is in respect to the carrageenan group. Statistical analysis one-way ANOVA, post hoc SNK test (*p* ≤ 0.05). ^b^At 5 h. ^c^Calculated using Molinspiration property engine v2018.10b [31]. ^d^Vs control carrageenan. ^e^Vs indomethacin; *n* = 5.

Following this, the acute toxicity of **3**–**6** was determined through one oral administration of 50 or 100 mg/kg in BALB/c mice, and after 14 days, no significant weight loss or lethality was observed in the individuals. Additionally, the post-mortem inspection of the kidneys, heart, and bowel of the experimental mice did not show any significant weight differences to the control group (Supporting Information File 1, Table 1, and Table 2).

Lastly, in order to acknowledge a potential mechanism of action of the bisphosphonates **3**–**6**, we propose that the tested derivatives are acting as MMP inhibitors. In this respect, MMP-8 and MMP-9 isoenzymes are related to inflammatory processes in different tissues [32–35]. Furthermore, for MMP-8 and MMP-9, enzyme–inhibitor interaction modes are well known. For example, the coordination of the P=O oxygen atom in bisphosphonates with a zinc cation in the catalytic site of the MMPs has been characterized, both through X-ray diffraction and molecular docking studies [11,36–37]. Consequently, we propose MMP-8 and MMP-9 as potential biological targets of **3**–**6**.

### Computational and theoretical analysis

#### Ligands structure–activity relationship

As a first approximation, we studied the structural and physicochemical features of the compounds to explain the antiinflammatory activity. The structure geometry used to obtain the molecular properties of each bisphosphonate represented a minimum in the potential energy surface since all vibrational frequency values were positive. In Table 3, all the molecular properties obtained for the compounds are displayed. The chemical hardness (η) and softness (*S*) were calculated using Equation 1 and Equation 2, which are based in the Koopman’s theorem for the determination of the global chemical reactivity descriptors.

[1] $$\eta =\frac{E_{\text{HOMO}}-E_{\text{LUMO}}}{2}$$

[2] $$S=\frac{1}{\eta }$$

**Table 3:** Molecular properties of the compounds **3**–**6**.

molecular properties	**3**	**4**	**5**	**6**

molecular weight (amu)	374.307	402.361	436.378	466.404
dipole moment (Debye)	3.4	3.4	2.43	2.4
*E*_HOMO_ (eV)	−10.57	−10.44	−9.41	−8.83
*E*_LUMO_ (eV)	0.82	0.72	0.25	0.19
volume (Å^3^)	363.78	401.16	430	455.41
PSA (Å^2^)	71.532	73.573	70.905	74.198
hardness (η)	5.69	5.58	4.83	4.51
softness (*S*)	0.1756	0.1792	0.207	0.2217

From Table 3, we observed that a correlation between the antiinflammatory activity of the compounds and the molecular properties can be established. The structural modifications are directly correlated to the molecular weight (MW) of the compounds, and the MW can be correlated to the molecular volume. We can appreciate that **3** and **4** have the lowest volume compared to **5** and **6**. This fact can help us to explain the greater antiinflammatory activity of **3** and **4** with a carrageenan model; a small molecular volume increases the pharmacokinetic abilities of the compounds. In addition, **3** and **4** have a greater dipole moment compared to **5** and **6**. The hydrogen-bond formation and the noncovalent interactions are influenced by the dipole moment. This means that an increased dipole moment can improve the binding properties of a molecule. Besides the dipole moment, **3** and **4** have the greatest chemical hardness of the bisphosphonates. This descriptor is related to the chemical susceptibility to an external potential. Therefore, the antiinflammatory activity of these compounds can be related to the size (volume), solvation (dipole moment), and chemical reactivity (hardness; probably related to a minor metabolic biotransformation). These descriptors can be associated with the better pharmacokinetic profile of the derivatives **3** and **4** by oral administration.

#### Molecular docking

As a second approximation, to study the effect of these structural modification on the pharmacodynamics, we performed a molecular docking over two MPPs. In Table 4, the interaction energy value (MolDock Score) [38] of each compound with the two different MMPs obtained from the docking calculation is displayed. Also, the ligand efficiency (LE) of each bisphosphonate is shown; the ligand efficiency stands for the coefficient of the interaction energy by the number of atoms in the molecule (excluding hydrogen atoms).

**Table 4:** MolDock Score and LE1 values (kcal/mol) for the docking experiments of the molecules **3**–**6** with MMP-8 and MMP-9 enzymes and the corresponding inhibition values.^a^

ligand	MMP-8		MMP-9		% inhibition
	MolDock Score	LE	MolDock Score	LE	

**3**	−146.72	−6.38	−134.16	−5.83	24.57
**4**	−137.92	−5.52	−147.76	−5.91	20.86
**5**	−150.42	−5.37	−147.24	−5.26	13.80
**6**	−168.58	−5.62	−146.50	−4.88	9.06

^a^Indomethacin was set as the reference (with 36.1272%).

By the inspection of the results above, there is a correlation between the LE parameter in MMP-8 and the experimental activity of the bisphosphonates, resulting in the highest LE value for **3**, with the most potent inhibition observed with the carrageenan model (Table 2). The predicted LE value for **4** in MMP-9 was comparable to **3** but showed less activity than **3**. For both dockings, **6** showed some of the highest MolDock Score values, but nevertheless, its inhibition activity was the lowest (Table 2). It is important to note that a clear correlation between the predicted interaction energy of **3**–**6** with MMP-8 and the topical antiinflammatory activity was observed (TPA model), with the derivative **6** being the most active one, followed by **5**, **3**, and **4** in that order (Table 1). Thus, the efficacy of the tested compounds **3**–**6** was well correlated with the lipophilicity and the predicted interaction energy with MMP-8 (Table 4). Next, we searched for other energy interaction features from the docking calculation.

**Figure 3:**
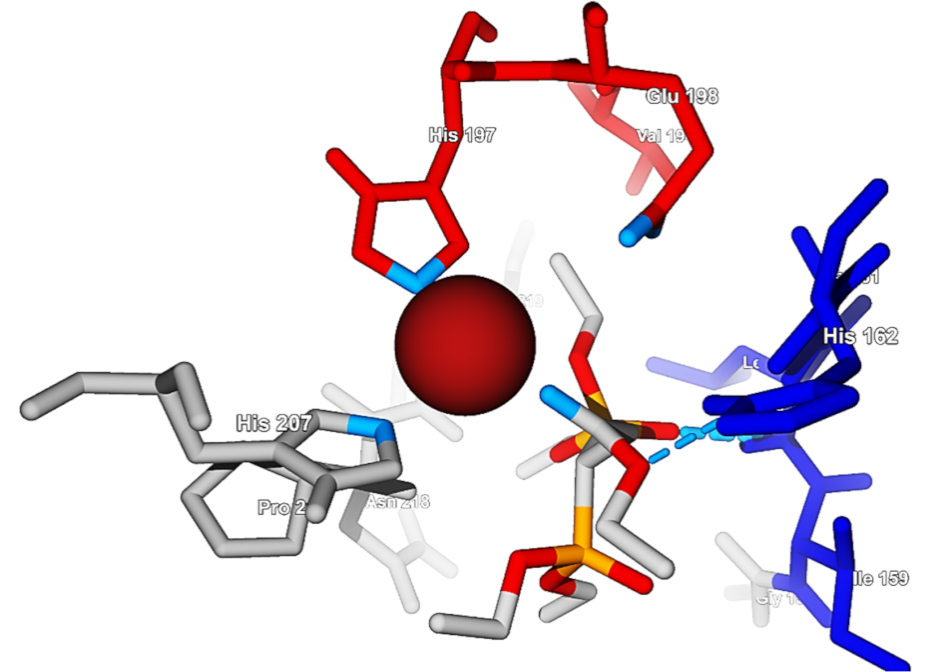
Coordination of the Zn^2+^ ion by residues and by the carbonyl ester oxygen atom of molecule **3**. The basic coordinating nitrogen and oxygen atoms are marked in light blue.

As expected, **4**–**6** could bind the Zn^2+^ ion in a monodentate fashion through the oxygen atom double bonded to the phosphorus atom (P=O) of one of the phosphonate moieties, as reported in the literature for other structures [38–39]. Only for **3**, zinc chelation was observed through the oxygen atom double bonded to the carbon atom (C=O) of the ester group (Figure 3). In all cases, a distorted tetrahedral coordination geometry was observed for the zinc ion, caused by the chelation with His197, Glu198, and His207 residues. His201 was far apart from the catalytic site, and the basic nitrogen atom in this residue was pointing away from the zinc ion [39]. The calculated distances from the Zn^2+^ ion and the different sites at the ligand and the protein are summarized in Table 5.

**Table 5:** Calculated distances (Å) for the coordination of the zinc(II) ion at MMP-8.

molecule	**3**	**4**	**5**	**6**

His207	2.28	3.30	2.36	2.26
His197	2.47	2.46	2.45	2.44
Glu198	3.84	3.85	4.16	4.13
oxygen (ligand)	1.99	1.99	1.99	1.99

The zinc chelation in MMP-9 occurred in a different way. The predicted orientation of the histidine residues at the catalytic site were not aiming directly at the metal ion. However, it can be assumed that because of the flexibility of the protein in solution, the coordination to the metal ion would be possible. The targets **3** and **4** could coordinate the Zn^2+^ ion through the oxygen atom double bonded to the phosphorus atom (P=O) of one of the phosphonate moieties, while **5** and **6** had interactions through the C=O oxygen atom of the ester group. Because the interactions of the benzyl group of **5** and **6**, respectively, with the Phe110 residue present at the catalytic site through π–π interactions are possible, the orientation of the molecules inside the catalytic site allowed the coordination through the ester groups rather than through the phosphonate moieties. The calculated distances from the Zn^2+^ ion and the different sites at the ligand and the protein are summarized in Table 6.

**Table 6:** Calculated distances (Å) for the coordination of the zinc(II) ion at MMP-9.

molecule	**3**	**4**	**5**	**6**

His411	2.35	2.67	2.80	2.72
His401	2.72	3.33	3.30	3.17
Glu402	4.53	4.51	4.12	4.48
oxygen (ligand)	2.00	2.00	2.00	2.00

**Figure 4:**
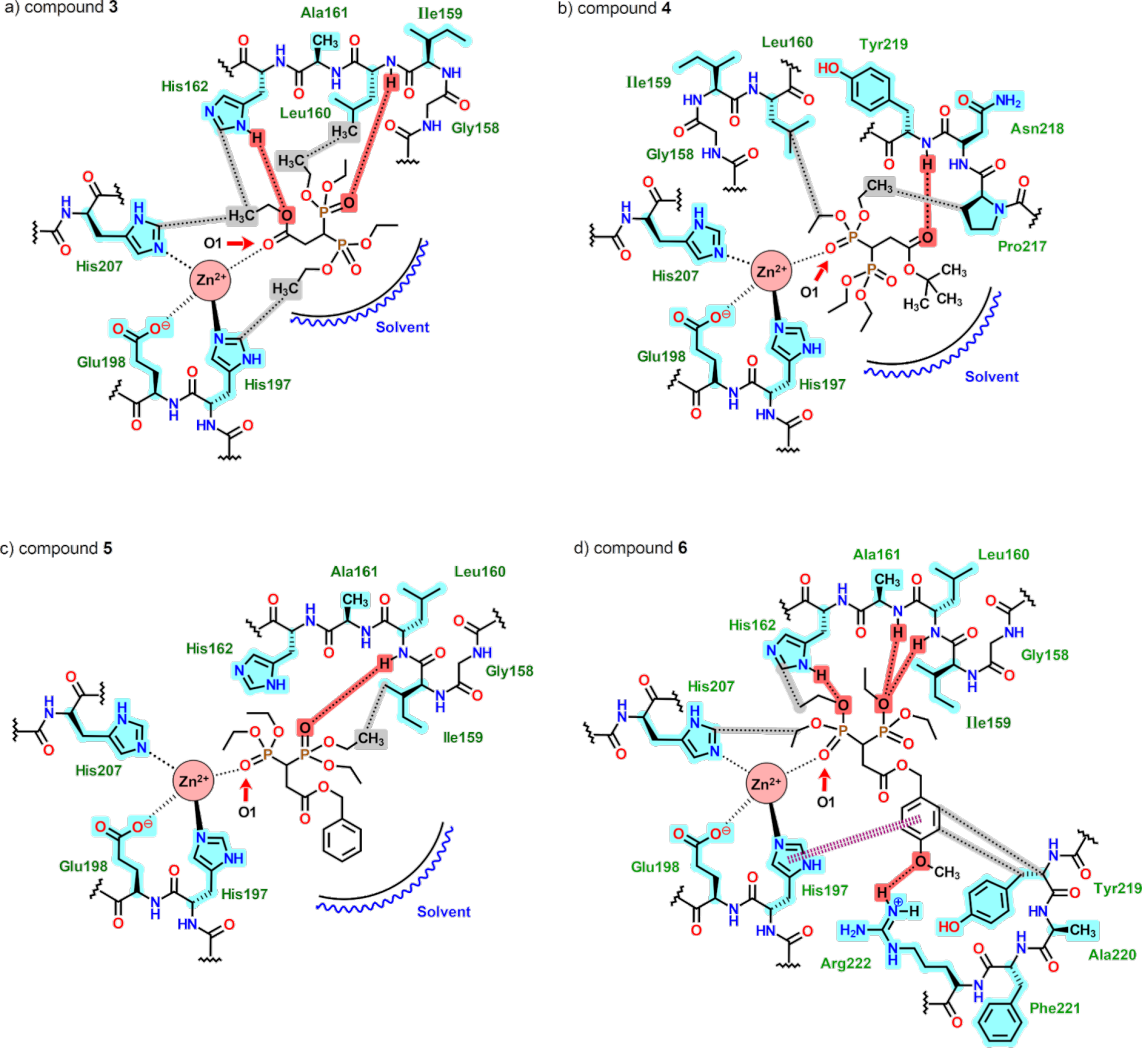
2D schematic representations of the MMP-8 catalytic site, with **3**–**6** and the most relevant interactions. Red = hydrogen bonds; dotted bonds = zinc complexation; highlighted in blue = most relevant residues; dashed pink lines = π–π interactions; grey = hydrophobic interactions.

In Figure 4, a schematic representation of the interactions of the ligands in MMP-8 is shown. As expected, the oxygen atoms that are coordinating the zinc ion have the greatest contribution. For example, **3** in MMP-8 has a greater contribution energy from oxygen O1, with an electrostatic energy (*E*_Elec_) of −24.20 kcal/mol and a total energy (*E*_Total_) of −22.03 kcal/mol; the observed energy values are shown in Table 7.

**Table 7:** Calculated total (*E*_Total_) and electrostatic energy (*E*_Elec_) for the most contributing oxygen atoms to the interaction energy of the complex. Energies in kcal/mol.

energy	MMP-8			
	**3**	**4**	**5**	**6**

*E*_Total_ (O1)	−22.03	−32.22	−35.880	−34.11
*E*_Elec_ (O1)	−24.20	−39.97	−38.200	−38.71
*E*_Elec_ (Zn)	−13.63	−22.83	−20.4092	−19.51

Compound **3** displayed two hydrogen bonds. The first one was formed between a hydrogen atom of His162 with an oxygen atom from the ester moiety (the oxygen atom bound to the ethyl group of the phosphonate unit), with a calculated distance (O–H) of 1.86 Å (or a 2.60 Å O–N distance) and an energy of −1.51 kcal/mol. The second interaction, stronger than the first one, was observed from an oxygen atom (P=O) of the phosphonate unit to an amide hydrogen atom located between Ile159 and Leu160. It possesses an energy of −2.5 kcal/mol and a 1.86 Å distance from the oxygen atom to the amide hydrogen atom (or a 3.09 Å O–N distance, Figure 4a). Compound **4** only displayed one hydrogen bond of the double bonded carbonyl oxygen atom to an amide hydrogen atom located between Asn218 and Tyr219. This interaction was regarded as weak, with an energy of −0.47 kcal/mol and a 3.51 Å O–N distance (Figure 4b). Also, for compound **5**, only one hydrogen bond interaction was seen. Indeed, the oxygen atom double bonded to the phosphorus atom of the phosphonate group was strongly bonded to an amide hydrogen atom localized between Ile159 and Leu160, with a 3.08 Å O–N distance and an energy of −2.5 kcal/mol (Figure 4c). Compound **6** showed four hydrogen bonds that varied in force. Two were observed between an oxygen atom from an OEt moiety of a phosphonate group to amide hydrogen atoms localized between Ile159, Leu160, and Ala161 (Figure 4d). These interactions had energy values of −1.94 and −2.5 kcal/mol, which is regarded as characteristic for strong hydrogen bonds. The measured O–N distances had values of 3.15 and 2.67 Å. Another hydrogen-bond interaction was seen from an OEt moiety (from the phosphonate group coordinating the zinc ion), with a weak energy of −0.95 kcal/mol and O–N distance of 3.26 Å. Finally, the methoxy group at the ligand had a weak energy of −1.0 kcal/mol and a 3.12 Å distance (Figure 4d).

**Figure 5:**
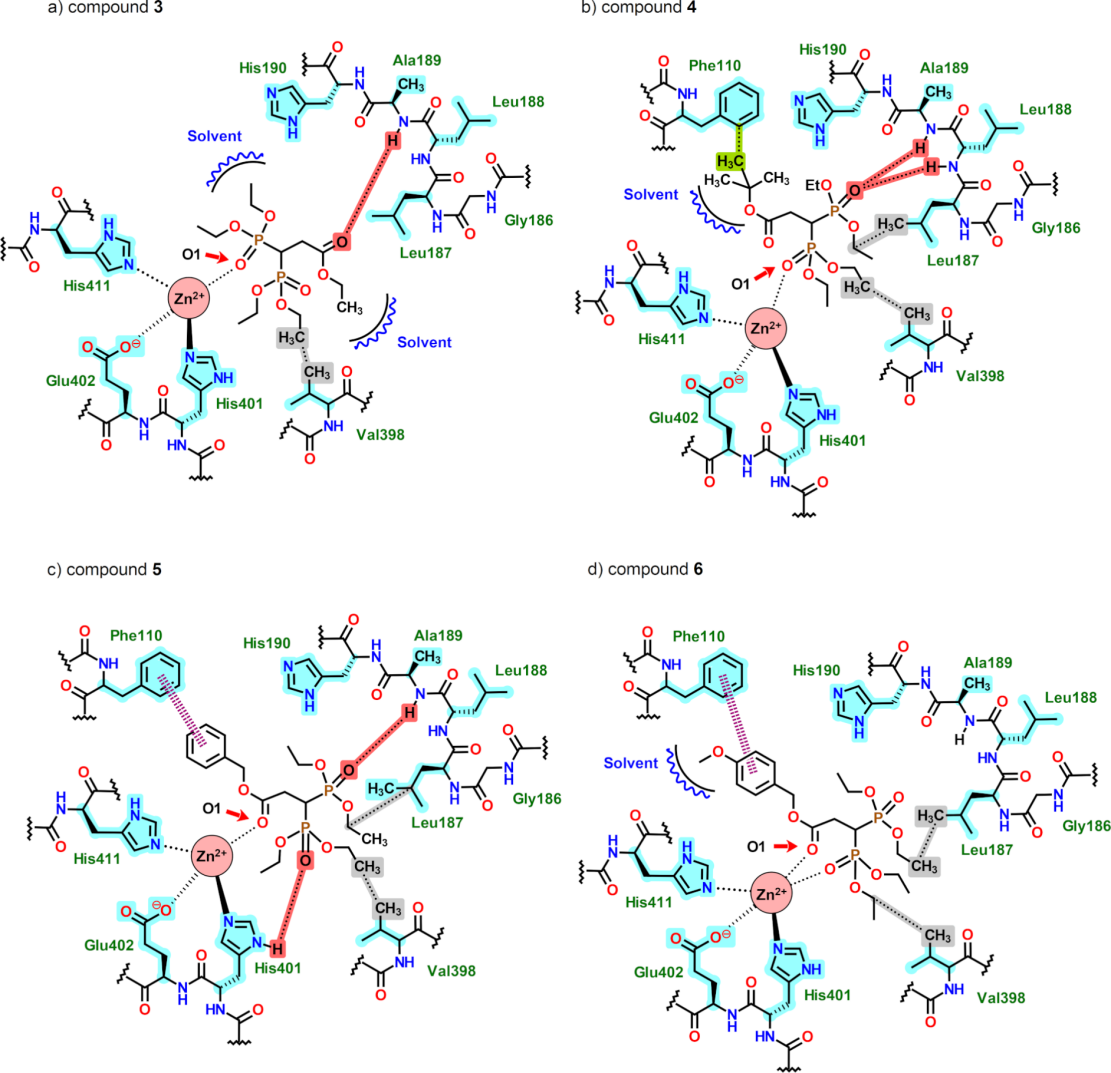
2D schematic representations of the MMP-9 catalytic site, with **3**–**6** and the most relevant interactions. Red = hydrogen bonds; dotted bonds = zinc complexation; highlighted in blue = most relevant residues; dashed pink lines = π–π interactions; grey = hydrophobic interactions; green = CH–π interactions.

As can be seen in Figure 4, the hydrophobic zones of the catalytic site in MMP-8 were formed by Ile159, Leu160, and Ala161, as well for Gly158 and Val194. However, for **3**, the ethyl groups were not entirely pointing at this zone. The calculated C–C distance from a methylene group to a Leu160 methyl group was 3.35 Å. The other methyl groups from the ligand had interactions with His162 (2.96 Å), His197 (2.98 Å), and His207 (3.20 Å). Indeed, the rest of the molecules also presented this type of interactions between an ethyl fragment of the ligand with Leu160, and less with His207 and Val194. Weak hydrophobic interactions were also observed from Tyr219 and the aromatic ring of **6** as well as π–π interactions to His197. The molecules were not adequately occupying the cavity of the enzyme in MMP-8, as shown in Figure 4. For the compounds **4** and **5**, the *tert*-butyl and benzyl groups at the ester moiety were pointing out to the solvent, without any important interactions with the hydrophobic zone inside the cavity (Figure 4b and Figure 4c). The only molecule that was occupying the entire pocket was compound **6** due to the formation of four hydrogen bonds and a favourable π–π interaction between the methoxybenzyl group and His197.

Only for **3** and **4**, one of the oxygen atoms of the phosphonate units was the most contributing atom to the interaction energy. However, for **5** and **6**, the electrostatic energy was almost equally distributed in the three oxygen atoms of the two phosphonate and ester groups. For example, in **5**, the three above-mentioned oxygen atoms displayed a total energy-per-atom value of −17.26 (C=O), −15.62 (P1=O), and −14.07 (P2=O) kcal/mol, although the electrostatic energy (*E*_Elec_) was higher at the ester oxygen atom (C=O), with a value of −23.95 kcal/mol, while the other two oxygen atoms from the phosphonate units had energies of −13.65 and −11.53 kcal/mol, respectively. The same energy trend was observed for **6**, which correlated well with the predicted interactions of the molecule (the C=O and P=O units binding Zn^2+^) at the catalytic site (Table 8).

**Table 8:** Calculated total (*E*_Total_) and electrostatic energy (*E*_Elec_) for the most contributing oxygen atoms to the system. Energies in kcal/mol.

energy	MMP-9			
	**3**	**4**	**5**	**6**

*E*_Total_ (O1)	−38.4103	−39.9732	−23.9506	−23.8484
*E*_Elec_ (O1)	−34.1810	−32.8937	−17.2600	−7.5905
*E*_Elec_ (Zn)	−20.9699	−19.8742	−20.2706	−19.022

As before, in Figure 5, the schematic representations of the interaction of the ligands in MMP-9 are displayed. Compound **3** displayed one hydrogen bond, from the carbonyl oxygen atom to an amide hydrogen atom located between Ala189 and Leu188, with this interaction being regarded as very weak, having an energy of −0.36 kcal/mol and a 3.53 Å O–N distance (Figure 5a). On the other hand, **4** had two hydrogen bond interactions from an oxygen atom (P=O) of the phosphonate group to amide hydrogen atoms located between Ala189, Leu188, and Leu187, with energy values of −2.36 and −1.60 kcal/mol (Figure 5b). Compound **5** exhibited two weak hydrogen bonds from an OEt oxygen atom to an amide hydrogen atom of Ala189 and Leu188 (Figure 5c). The energy value was −0.89 kcal/mol, and the O–N distance was 3.24 Å. The second interaction was very weak, with a value of −0.15 kcal/mol from a P=O oxygen atom to the His401 amine hydrogen atom. Finally, for **6**, the hydrogen bond interactions were lacking. From the energy contribution profile seen in Figure 5d, most of the interaction energy was due to the coordination of the ligand to the zinc ion, with the same applying to **5**. This could explain why no predicted hydrogen bonding was present in **6** and just very weak ones in **5**.

For MMP-9, the hydrophobic zones were formed by Ile159, Leu160, and Ala161, as was the case for Gly158 and Val194. As stated before, π–π interactions from the aromatic ring of the ligands **5** and **6** with the Phe110 residue were displayed (Figure 5c and 5d). Furthermore, CH–π interactions from the *tert*-butyl group with Phe110 were also seen, with a 3.12 Å distance (Figure 5b). All molecules had hydrophobic interactions from an ethyl group of the ligands to a methyl moiety of Val398 and with Leu187, with distances below 3.5 Å.

For MMP-9, the ligands were even more exposed to the solvent, although many more noncovalent interactions were seen between the catalytic site and the molecules (Figure 5).

## Conclusion

In this work, we reported the two-step synthesis of the bisphosphonic esters **3**–**6**. For the first time, the antiinflammatory activity of the compounds was assessed by oral (carrageenan model) and topical administration (TPA model) to mice. Among these, the derivative **6** had an excellent edema inhibition, comparable to the positive control with the TPA model. On the other hand, the bioisosteric replacement of an amide for an ester group in the parent compounds **1** and **2** afforded the more potent derivatives **3** and **4**, which had a higher antiinflammatory activity than the parent bisphosphonates **1** and **2** using a carrageenan model. Moreover, a lipophilicity–activity relationship was observed for the two acute inflammation models: the aliphatic hydrophilic compounds **3** and **4** were the more potent ones by oral administration, and the aromatic lipophilic bisphosphonates **5** and **6** had a better antiinflammatory activity by topical administration. In addition, the safety of the test compounds **3**–**6** was evaluated by an acute toxicity determination where no significant weight loss or lethality was observed in individuals at a 50 and 100 mg/kg dose (the two- or four-fold dose as used in the original study). Finally, a ligand structure–activity relationship and molecular docking analysis led us to propose MMP-8 and MMP-9 inhibition as the possible action mechanism of **3**–**6** due to the good correlation between the antiinflammatory activity of the bisphosphonic esters and the interaction energy with these enzymes (especially MMP-8). Also, a good correlation between the biological effects and interaction of the compounds with the Zn^2+^ cofactor of these enzymes was observed.

## Supporting Information

File 1 General procedures of the synthesis, characterization of the compounds, the biological activity methodology, computational details, and NMR/HRMS spectra of the final products.
