# Supplementary material for: Synthesis, antiinflammatory activity, and molecular docking studies of bisphosphonic esters as potential MMP-8 and MMP-9 inhibitors
Source: Beilstein J Org Chem. 2020 Jun 8;16:1277–87. doi: 10.3762/bjoc.16.108 (PMC7296197; doi:10.3762/bjoc.16.108)
Supplement: File 1 — General procedures of the synthesis, characterization of the compounds, the biological activity methodology, computational details, and NMR/HRMS spectra of the final products. [file Beilstein_J_Org_Chem-16-1277-s001.pdf]

BEILSTEIN JOURNAL OF ORGANIC CHEMISTRY

Supporting Information

for

Synthesis, antiinflammatory activity, and molecular docking studies of bisphosphonic esters as potential MMP-8 and MMP-9 inhibitors

Abimelek Cortes-Pacheco, María Adelina Jiménez-Arellanes, Francisco José Palacios-Can, José Antonio Valcarcel-Gamiño, Rodrigo Said Razo-Hernández, María del Carmen Juárez-Vázquez, Adolfo López-Torres and Oscar Abelardo Ramírez-Marroquín

Beilstein J. Org. Chem. **2020,** *16,* 1277–1287. doi:10.3762/bjoc.16.108

General procedures of the synthesis, characterization of the compounds, the biological activity methodology, computational details, and NMR/HRMS spectra of the final products

License and Terms: This is a supporting information file under the terms of the Creative Commons Attribution License (http://creativecommons.org/licenses/by/4.0). Please note that the reuse, redistribution and reproduction in particular requires that the authors and source are credited.
The license is subject to the *Beilstein Journal of Organic Chemistry* terms and conditions: (https://www.beilstein-journals.org/bjoc)

Table of contents

▪ Instrumentation and chemicals	S2
▪ Experimental procedures of synthesis	S3
▪ Characterization data	S4
▪ In vivo antiinflammatory activity	S7
▪ Computational details	S9
▪ References	S10
▪ Spectral data (NMR and HRMS)	S11

Instrumentation and chemicals

All commercial materials were used as received from Sigma-Aldrich without further purification. The solvents were dried by standard techniques. TLC: Merck 0.2 mm silica gel 60 F_{254} analytic aluminum plates. Column chromatography (CC): 230–400-mesh Silica Flash 60® silica gel.

NMR spectra were measured with a Bruker 500 MHz (^{13}C: 125.8 MHz, ^{1}H: 500.6 MHz, ^{31}P: 200.7 MHz) and a Magritek Spinsolve 80 spectrometer (^{13}C: 20 MHz, ^{1}H: 80 MHz). ^{31}P NMR spectra were recorded with H_3PO_4 as an external reference. ^{1}H and ^{13}C NMR spectra were recorded with TMS as an internal standard in $CDCl_3$. Chemical shifts are reported in ppm. Multiplicities are recorded as follows: s = singlet, d = doublet, t = triplet, dd = doublet of doublets, td = triplet of doublets, bs = broad singlet, q = quartet, and m = multiplet. Coupling constants (*J*) are given in Hz. High-resolution mass spectra (HRMS), using electrospray ionization (ESI), were recorded on a SYNAPT G2-Si (Waters) spectrometer, equipped with a single quadrupole mass filter and a time-of flight-mass analyzer (Q-TOF).

Experimental procedures of synthesis

General procedure for the synthesis of the bromoacetoesters 7–10

Method A

A mixture of ethyl or *tert*-butyl alcohol (12 equiv) and triethylamine (1.2 equiv) was stirred in dry CH_2Cl_2 at 0 °C for 10 min, and then, bromoacetyl bromide (1.0 equiv) was added dropwise and stirred at 0–25 °C for 5 hours. Next, water (5 mL) was added and the mixture was extracted with CH_2Cl_2 (3×10 mL), the combined organic layer was dried over Na_2SO_4, and concentrated under vacuum, yielding the bromoacetoesters **7** and **8**.

Method B

To a stirred solution of sodium bicarbonate (3 equiv) and benzyl alcohol or 4-methoxybenzyl alcohol (1 equiv), bromoacetyl bromide (1.4 equiv) was added dropwise in dry acetonitrile at 0 °C. The mixture was stirred at room temperature for 5 h, then, water (5 mL) was added, and the mixture was extracted with CH_2Cl_2 (3×10 mL), the combined organic layer was dried over Na_2SO_4, and concentrated under vacuum to afford the bromoacetoesters **9** and **10**.

General procedure for the synthesis of the bisphosphonates 3–6

NaHMDS (1.5 equiv) was added dropwise to a solution of tetraethylmethylene disphosphonate in THF (10 mL) at −5 °C under nitrogen. After that, the respective alkyl halide **7**–**10** (2.5 equiv), respectively, was added at −5 °C to the previous solution. The reaction mixture was stirred at 0 °C for 18 h. Finally, the crude reaction was concentrated, and the crude product was purified by column chromatography (AcOEt:MeOH, 95:05), yielding the bisphosphonates **3**–**6**.

Characterization data for 3-6 and 7-10

Ethyl 2-bromoacetate (7, $C_4H_7BrO_2$)

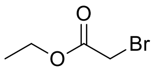

7

Yield: 0.4 g (42%) as a colorless oil. ^{1}H and ^{13}C NMR spectra are consistent with those reported in [1].

***tert*-Butyl 2-bromoacetate (8, $C_6H_{11}BrO_2$)**

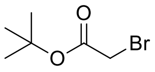

8

Yield: 0.78 g (71%) as a colorless oil. ^{1}H and ^{13}C NMR spectra are consistent with those reported in [2].

Benzyl 2-bromoacetate (9, $C_9H_9BrO_2$)

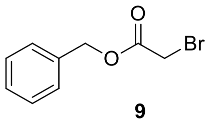

9

Yield: 0.66 g (71%) as a colorless oil. ^{1}H and ^{13}C NMR spectra are consistent with those reported in [3].

4-Methoxybenzyl 2-bromoacetate (10, $C_{10}H_{11}BrO_3$)

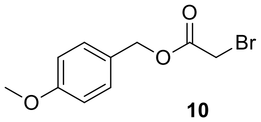

10

Yield: 0.92 g (91%) as a colorless oil. ^{1}H and ^{13}C NMR spectra are consistent with those reported in [4].

Ethyl 3,3-bis(diethoxyphosphoryl)propanoate (3, $C_{13}H_{28}O_8P_2$).

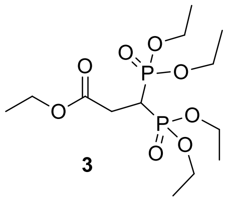

3

Yield: 0.44 g (73%) as a yellow viscous oil. **^{1}H NMR** (500 MHz, $CDCl_3$): δ = 1.25 (t, J = 7.2 Hz, 3H, CH_3), 1.33 (t, J = 7.1, 12H, CH_3), 2.81 (td, J = 15.9, 6.3 Hz, 2H, CH_2C=O), 3.08 (tt, J = 23.8, 6.2 Hz, 1H, CHP_2), 4.10 – 4.24 (m, 10H, OCH_2) ppm. **^{13}C NMR** (126 MHz, $CDCl_3$): δ = 14.13 (CH_3), 16.27 (d, J = 2.9 Hz, CH_3), 16.32 (d, J = 2.8 Hz, CH_3), 30.61 (t, J = 4.5 Hz, CH_2C=O), 32.82 (t, J = 136.0 Hz, CHP_2), 61.22 (OCH_2), 62.77 (d, J = 6.5 Hz, $POCH_2$), 62.92 (d, J = 6.5 Hz, $POCH_2$), 170.90 (C=O) ppm. **^{31}P NMR** (202 MHz, $CDCl_3$): δ = 22.23 ppm. **HRMS** [ESI$^+$]: *m/z* calculated 375.1338, found 375.1340.

***tert*-Butyl 3,3-bis(diethoxyphosphoryl)propanoate (4, $C_{15}H_{32}O_8P_2$).**

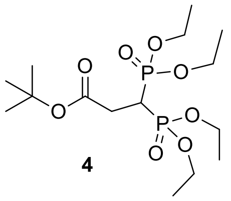

4

Yield: 0.48 g (44%) as a yellow viscous oil. **^{1}H NMR** (500 MHz, $CDCl_3$): δ = 1.33 (t, J = 7.1, 12H, CH_3), 1.46 (s, 9H, CH_3), 2.76 (td, J = 16.1, 6.0 Hz, 2H, CH_2C=O), 3.07 (tt, J = 23.9, 6.0 Hz, 1H, CHP_2), 4.12 – 4.23 (m, 8H, $POCH_2$) ppm. **^{13}C NMR** (126 MHz, $CDCl_3$): δ = 16.34 (d, J = 6.2 Hz, CH_3), 28.01 (CH_3), 31.44 (t, J = 4.4 Hz, CH_2C=O), 33.06 (t, J = 135.8 Hz, CHP_2), 62.70 (d, J = 6.5 Hz, $POCH_2$), 62.84 (d, J = 6.5 Hz, $POCH_2$), 81.31 (CCH_3), 169.85 (C=O) ppm. **^{31}P NMR** (202 MHz, $CDCl_3$): δ = 22.74 ppm. **HRMS** [ESI$^+$]: *m/z* calculated 403.1651, found 403.1650.

Benzyl 3,3-bis(diethoxyphosphoryl)propanoate (5, $C_{18}H_{30}O_8P_2$).

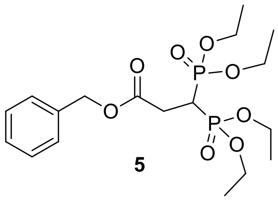

5

Yield: 0.56 g (65%) as a yellow viscous oil. **^{1}H NMR** (500 MHz, $CDCl_3$): δ = 1.32 (t, J = 6.9 Hz, 12H, CH_3), 2.91 (td, J = 15.8, 6.3 Hz, 2H, CH_2C=O), 3.13 (tt, J = 23.7, 6.3 Hz, 1H, CHP_2), 4.10-4.25 (m, 8H, $POCH_2$), 5.17 (s, 2H, CCH_2), 7.33-7.41 (m, 5H, H_{arom}) ppm. **^{13}C NMR** (126 MHz,

$CDCl_3$): δ = 16.32 (d, *J* = 6.3 Hz, CH_3), 16.29 (d, *J* = 6.2 Hz, CH_3), 30.67 (t, *J* = 4.5 Hz, CH_2C=O), 32.87 (t, *J* = 136.0 Hz, CHP_2), 62.82 (d, *J* = 6.6 Hz, $POCH_2$), 62.96 (d, *J* = 6.5 Hz, $POCH_2$), 67.03 (CCH_2), 128.34 (C_{arom}), 128.36 (C_{arom}), 128.56 (C_{arom}), 135.56 (C_{arom}), 170.76 (C=O) ppm. **^{31}P RMN** (202 MHz, $CDCl_3$): δ = 22.08 ppm. **HRMS** [ESI$^+$]: *m/z* calculated 437.1494, found 437.1505.

4-Methoxybenzyl 3,3-bis(diethoxyphosphoryl)propanoate (6, $C_{19}H_{32}O_9P_2$).

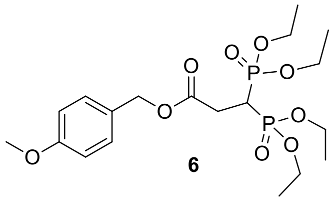

6

Yield: 0.36 g (41%) as a yellow viscous oil. **^{1}H NMR** (80 MHz, $CDCl_3$): δ = 1.34 (t, *J* = 7.3 Hz, 12H, CH_3), 2.62-3.47 (m, 3H, $CHCH_2$), 3.84 (s, 3H, CH_3), 4.50 – 3.89 (m, 8H, $POCH_2$), 5.12 (s, 2H, CCH_2), 6.91 (d, *J* = 8.6 Hz, 1H, H_{arom}), 7.34 (d, *J* = 8.9 Hz, 1H, H_{arom}) ppm. **^{13}C NMR** (20 MHz, $CDCl_3$): δ = 16.38 (d, *J* = 6.2 Hz, CH_3), 30.76 (t, *J* = 4.4 Hz, CH_2C=O), 32.95 (t, *J* = 135.9 Hz, CHP_2), 55.35 (CH_3O), 63.00 (d, *J* = 6.6 Hz, $POCH_2$), 62.86 (d, *J* = 6.6 Hz, $POCH_2$), 66.93 (CCH_2), 114.01 (C_{arom}), 127.78 (C_{arom}), 130.26 (C_{arom}), 159.83 (C_{arom}), 170.85 (C=O) ppm. **HRMS** [ESI$^+$]: *m/z* calculated 467.1600, found 467.1612.

In vivo antiinflammatory activity

Adult Balb/C mice (25 ± 3 g) were used for the antiinflammatory (male) and acute toxicity studies (female). Mice were obtained from the Animal Vivarium of CMN-SXXI, IMSS, Mexico City and were maintained in plastic cages during a 7-day conditioning period prior to the experiments under laboratory conditions (12 h/12 h light/dark cycles; temperature 25 ± 2 °C; humidity 55–80%) with rodent chow food and water ad libitum. The experiments were performed following the statutes of the International Committee for the Care and Use of Laboratory Animals (IACUC) and Mexican Official Norm (NOM-062-ZOO-1999) revised in 2015.

Carrageenan-induced edema in mice

This model was performed as described in [5]. Treated groups (n = 5) received by the intragastric route indomethacin (20 mg/kg) and the compounds **3**–**6** (25 mg/kg) 1 h prior to the injection of carrageenan (20 mL, 2%). The samples were solubilized in tween 80:H_2O, 1:9 and the control received only vehicle. The percentage of inhibition was calculated by comparing the measurement of the paw edema at different times (1, 2, 3, 5, and 7 h, E_t) using a digital micrometer and the value of time zero (baseline, E_0). The results were analyzed with the formula:

$$\% \text{ inhibition} = [(E_t - E_o) \text{ carrageenan} - (E_t - E_o) \text{ treated} / (E_t - E_o) \text{ carrageenan}] \cdot 100.$$

Tetradodecylphorbol-13-acetate (TPA)-induced ear edema

This assay was conducted as is described in [5]. The control was treated with TPA (2.5 μg) in acetone on the right ear (Ws) and then the left ear received only 25 mL of acetone (Wo). The experimental groups (n = 5) received TPA and 30 min later were treated with indomethacin (2 mg/ear) or compounds **3**–**6** (2 mg/ear) in the right ear (Ws). The antiinflammatory activity was calculated according to the weight difference between the ear sections (6 mm) at 6 h, compared with the control group, using the following formula:

$$\% \text{ Inhibition} = [(Ws - Wo) \text{ control} - (Ws - Wo) \text{ treated}/(Ws - Wo) \text{ control}] \cdot 100.$$

Acute toxicity

This test was performed according to the procedure TG 423 described by the Organization for Economic Cooperation and Development and Test Guideline (OECD) in [6]. Groups of five animals were employed, and compounds **3**–**6** were administered by an intragastric route after a fasting period of 12 h. The control received only the vehicle (Tween 80:H_2O, 1:9), and the treated group received a single administration of compounds **3**–**6** at 50 and 100 g/kg body weight (BW) doses, in a volume not exceeding 10 mL/kg BW. The animals were maintained under observation for 14 days and their

BW gain was recorded on days 3, 7, 9, and 14. After that, the animals were euthanized and the liver, spleen, and both kidneys were extracted to find relative weight changes.

Table 1. Effect of a single-dose administration (i.g.) of **3**–**6** on BW gain in female Balb/C mice.

treatment	days				deaths
	**3** (g)	**7** (g)	**9** (g)	**14** (g)	
control	0.006 ± 0.15	0.034 ± 0.17	0.262 ± 0.35	0.616 ± 0.27	0/5
**3** (50 mg/kg)	0.246 ± 0.11	0.248 ± 0.08	0.214 ± 0.06	0.502 ± 0.15	0/5
**3** (100 mg/kg)	-0.274 ± 0.10	0.044 ± 0.03	0.194 ± 0.21	0.498 ± 0.22[b]	0/5
**4** (50 mg/kg)	-0.092 ± 0.11	0.09 ± 0.08	0.158 ± 0.09	0.404 ± 0.17[b]	0/5
**4** (100 mg/kg)	-0.084 ± 0.08	0.252 ± 0.17	0.304 ± 0.17	0.658 ± 0.18[b]	0/5
**5** (50 mg/kg)	-0.214 ± 0.12	0.044 ± 0.11	0.056 ± 0.04	0.606 ± 0.10[b,c,d]	0/5
**5** (100 mg/kg)	-0.386 ± 0.05[a]	0.028 ± 0.11	0.144 ± 0.15	0.706 ± 0.18[b,c,d]	0/5
**6** (50 mg/kg)	-0.232 ± 0.08	0.02 ± 0.05	0.108 ± 0.09	0.642 ± 0.13[b,c,d]	0/5
**6** (100 mg/kg)	0.188 ± 0.62	0.376 ± 0.67	0.654 ± 0.55	0.888 ± 0.62	0/5

Data presented as mean ± standard error (s.e.). Statistical analysis two-way ANOVA, post hoc SNK test ($p \leq 0.05$). Treatments: [a]vs control group; [b]vs day 3; [c]vs day 7; [d]vs day 9. $n = 5$.

Table 2. Effect of a single-dose administration (i.g.) of **3**–**6** on organ weights (g) in female Balb/C mice.

treatment	organ weight (g)		
	liver	spleen	kidney
**Control**	1.219±0.040	0.1732±0.002	0.2930±0.005
**3** (50 mg/kg)	1.2452±0.080	0.2336±0.010[a]	0.2983±0.010
**3** (100 mg/kg	1.1021±0.080	0.1804±0.010	0.2750±0.010
**4** (50 mg/kg)	1.0976±0.050	0.2089±0.007	0.3131±0.010
**4** (100 mg/kg)	1.2388±0.040	0.1991±0.007	0.3141±0.010
**5** (50 mg/kg)	1.0504±0.040[a]	0.2026±0.010	0.2809±0.390
**5** (100 mg/kg)	1.1283±0.040	0.1724±0.010	0.2938±0.390
**6** (50 mg/kg)	1.1256±0.030	0.2516±0.010[a]	0.3230±0.007[a]
**6** (100 mg/kg)	1.1822±0.060	0.2081±0.008	0.3271±0.010[a]

After 14 days, the mice were sacrificed, various organs dissected out, and immediately weighted. Data presented as mean ± standard error (s.e.). Statistical analysis one-way ANOVA, post hoc SNK test ($p \leq 0.05$). Treatments: [a]vs control group. $n = 5$.

Computational details

Computational details

Conformational analysis and geometry optimization

A systematic conformational analysis was performed to obtain the most stable conformer of each of the four bisphosphonic esters using the MMFFaq force field. An equilibrium geometry optimization was done without symmetric restrictions using the semiempirical quantum mechanic level of theory with the Parametric Method 6 (PM6) approximation. To validate the structure as a minimum in the potential energy surface, we performed a vibrational frequencies analysis. All the calculations were carried out using Spartan'18.

Molecular docking

All synthesized bisphosphonic esters as well as the cocrystallized ligands were docked into the catalytic site of the matrix metalloproteases MMP-8 (pdb 4QKZ) and MMP-9 (pdb 2OW1). The electrostatic partial charges type was used for all the ligands in this study. The search algorithm used was MolDock SE with a number of 15 runs, 3000 iterations, and a population of 100. MolDock Score GRID was used as a scoring function with a radius of 9 Å for the search sphere. For both metalloproteinases, flexible dockings were performed using the above-mentioned parameters. All the residues with a distance of 3 Å from the catalytic site centre were set as flexible, for this purpose, only residues with 3 or more torsions were considered. The validation of the methodology for the docking experiments was performed first by reproducing the cocrystallized ligand conformation in both MMP-8 and MMP-9. For the MMP-8 co-crystallized ligand, a RMSD value of 0.539 was obtained, and for the cocrystallized ligand of MMP-9, a RMSD value of 0.348. The docking studies were carried out using Molegro Virtual Docker.

References

1. Van der Klei, A.; de Jong, R. L.; Lugtenburg, J.; Tielens, A. G. *European Journal of Organic Chemistry*, **2002**, *17*, 3015-3023.
2. Siebum, A. H.; Woo, W. S.; Lugtenburg, J. *European Journal of Organic Chemistry*, **2003**, *23*, 4664-4678.
3. Ideue, E.; Toma, T.; Shimokawa, J.; Fukuyama, T. *Organic Syntheses*, **2003**, *89*, 501-509.
4. Yoshinaga, Y.; Yamamoto, T.; Suginome, M. *ACS Macro Lett.* **2017**, *6*, 705–710.
5. Pérez-González, M. Z.; Gutiérrez-Rebolledo, G. A.; Yépez-Mulia, L.; Rojas-Tomé I. S.; Luna-Herrera, J.; Jiménez-Arellanes, M. A. *Biomed. Pharmacother.* **2017**, *89*, 89–97.
6. *OECD. OECD 423- GUIDELINES FOR TESTING OF CHEMICALS: Acute Oral Toxicity - Acute Toxic Class Method; 2004*.

Spectral data (NMR and HRMS)

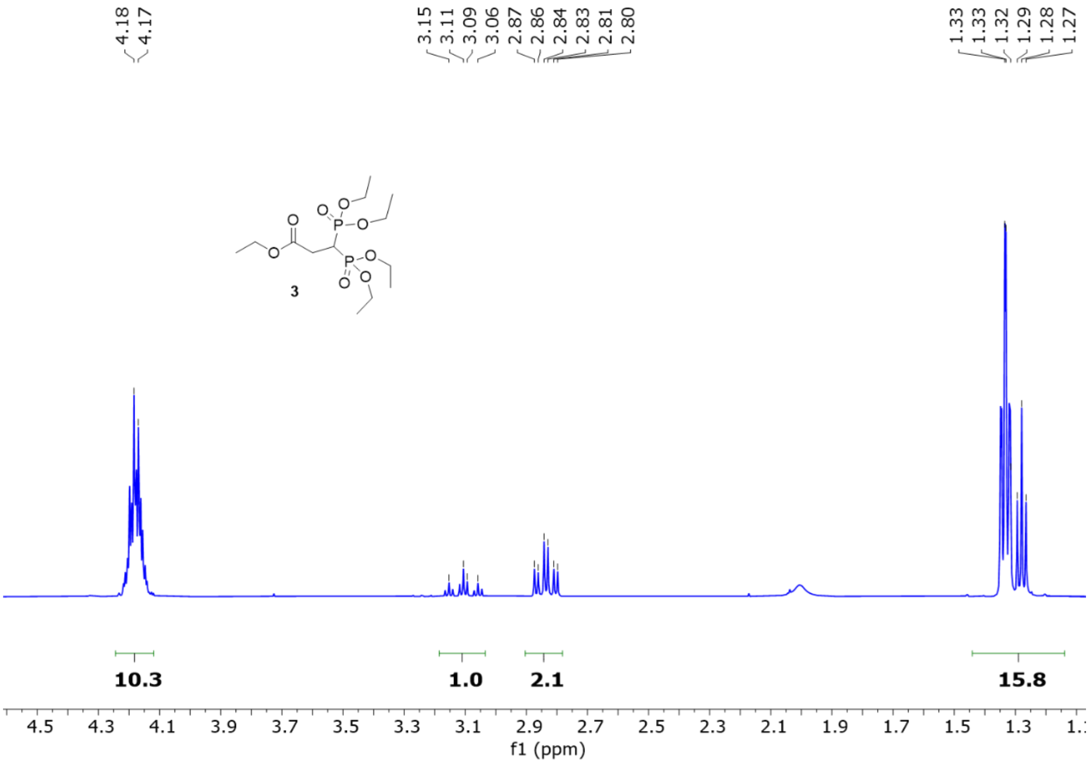

Fig. 1 ^{1}H NMR spectra of bisphosphonate **3**

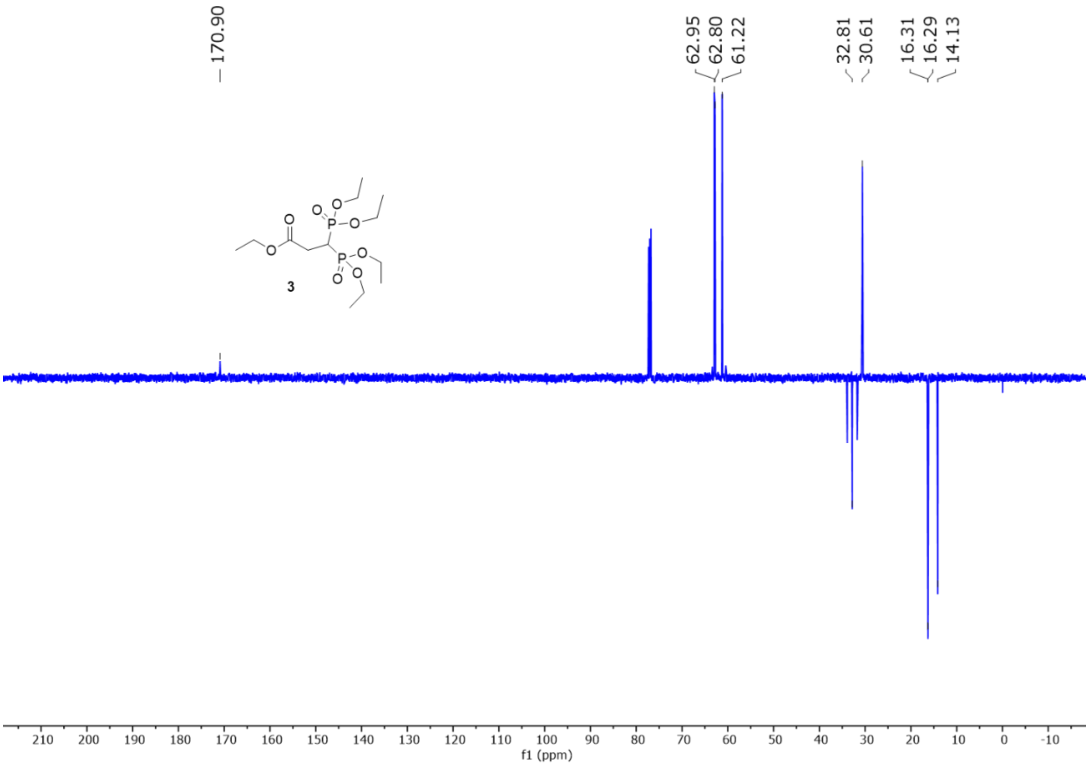

Fig. 2 DEPT ^{13}C NMR spectra of bisphosphonate **3**

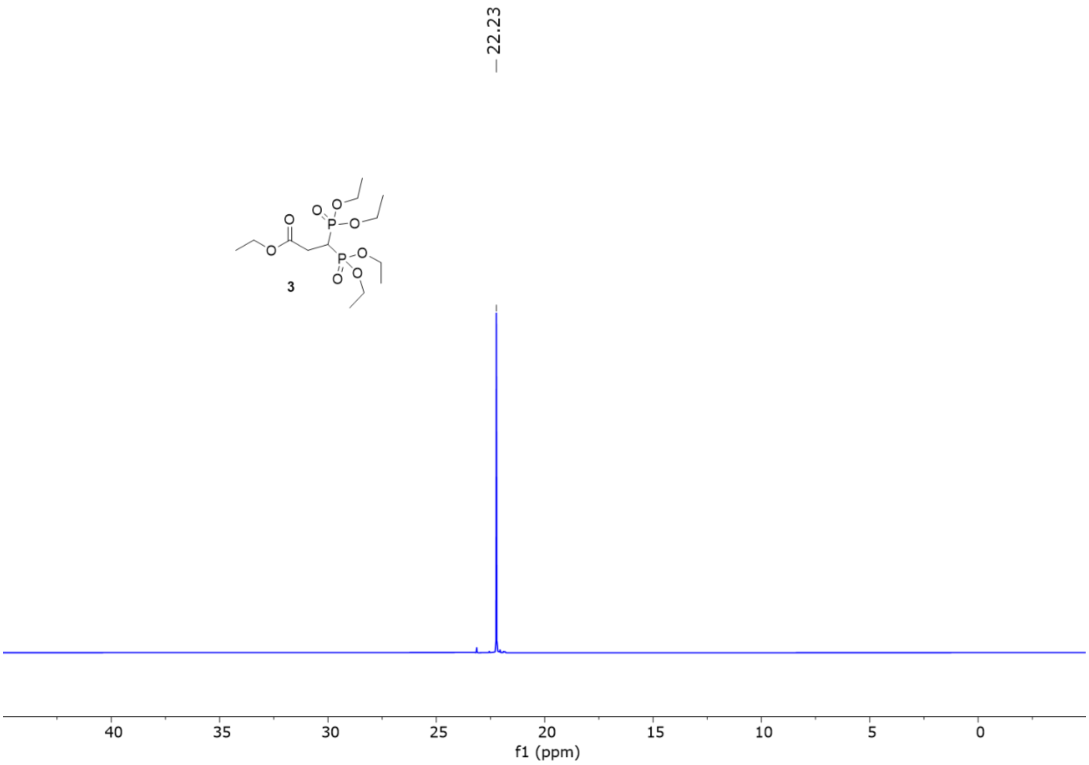

Fig. 3 ^{31}P NMR spectra of bisphosphonate **3**

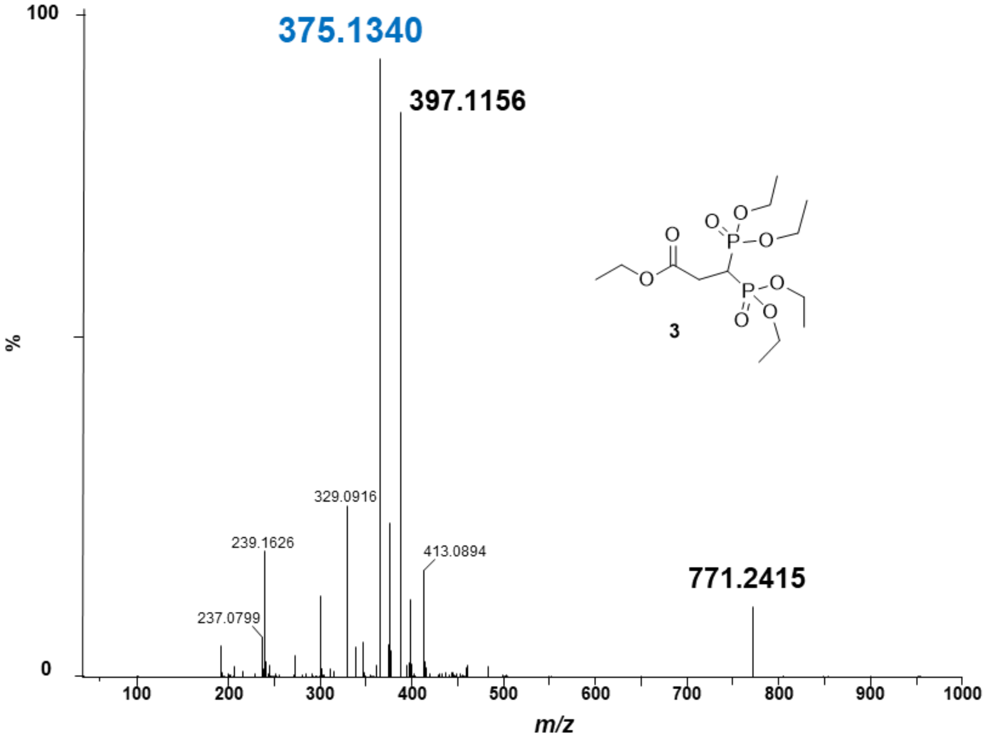

Fig. 4 HRMS spectra of bisphosphonate **3**

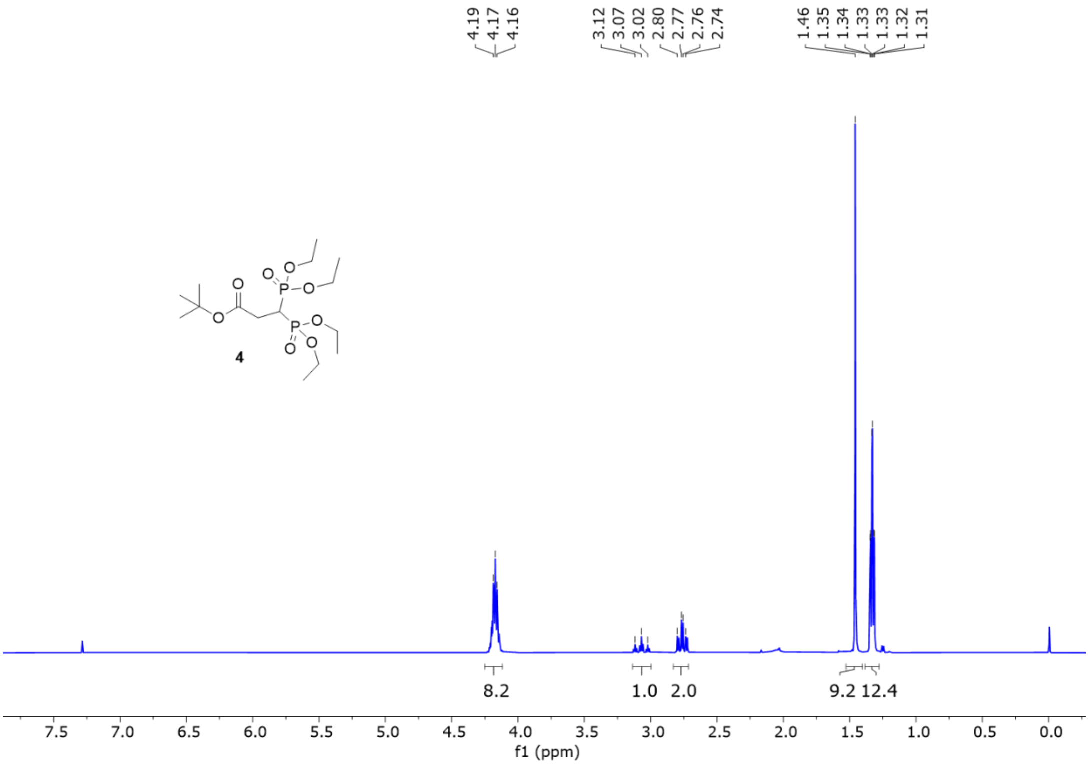

Fig. 5 ^{1}H NMR spectra of bisphosphonate **4**

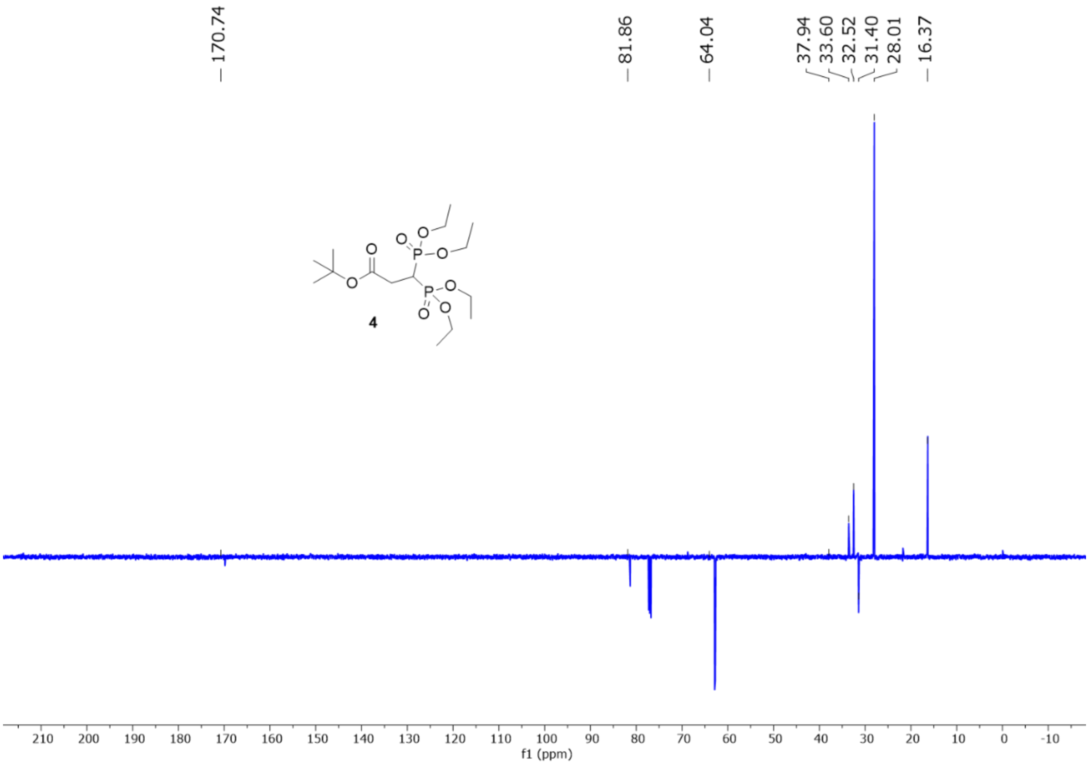

Fig. 6 DEPT ^{13}C NMR spectra of bisphosphonate **4**

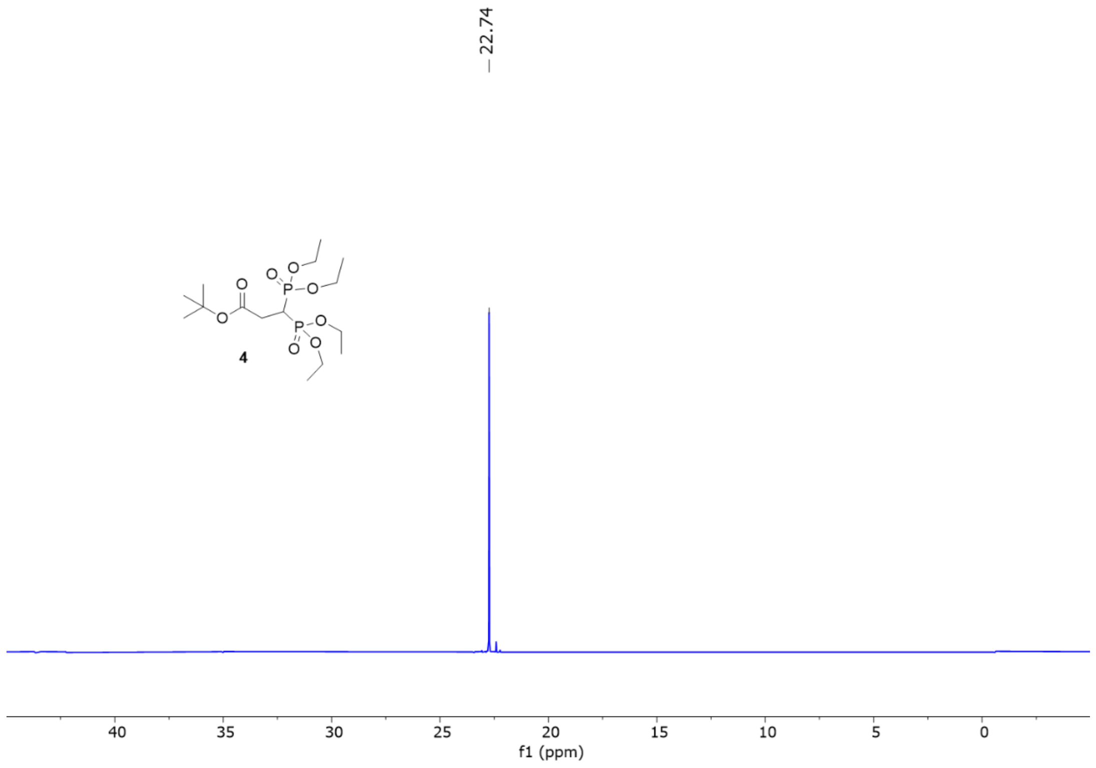

Fig. 7 ^{31}P NMR spectra of bisphosphonate **4**

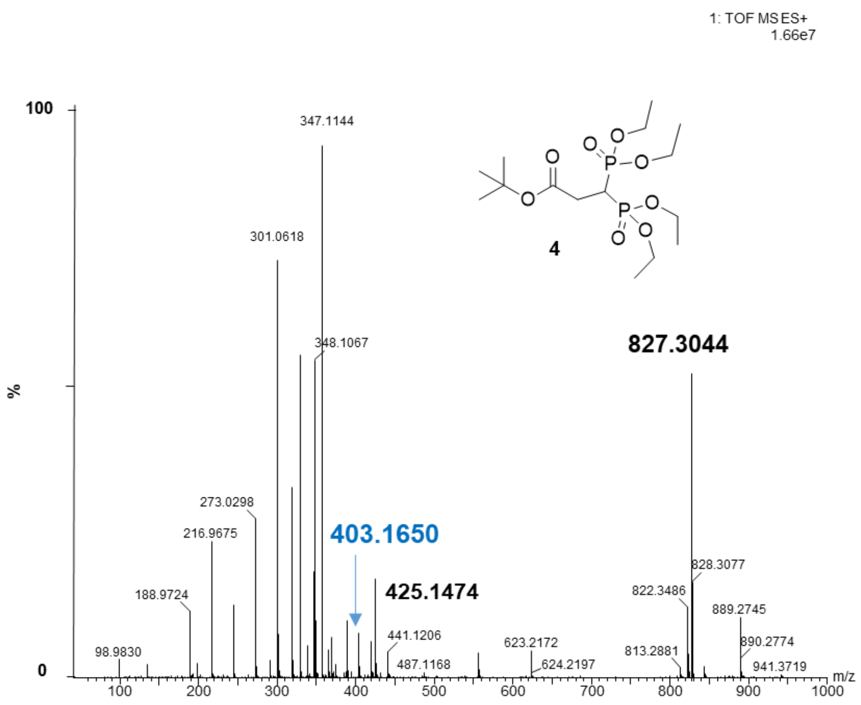

Fig. 8 HRMS spectra of bisphosphonate **4**

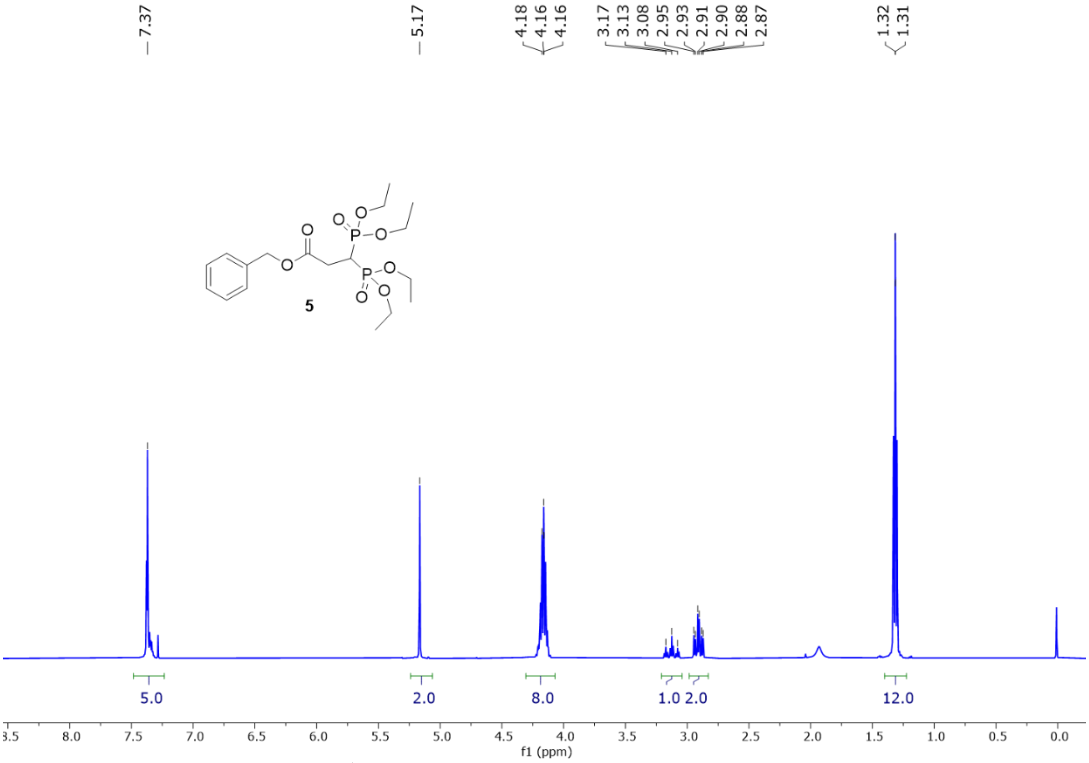

Fig. 9 ^{1}H NMR spectra of bisphosphonate **5**

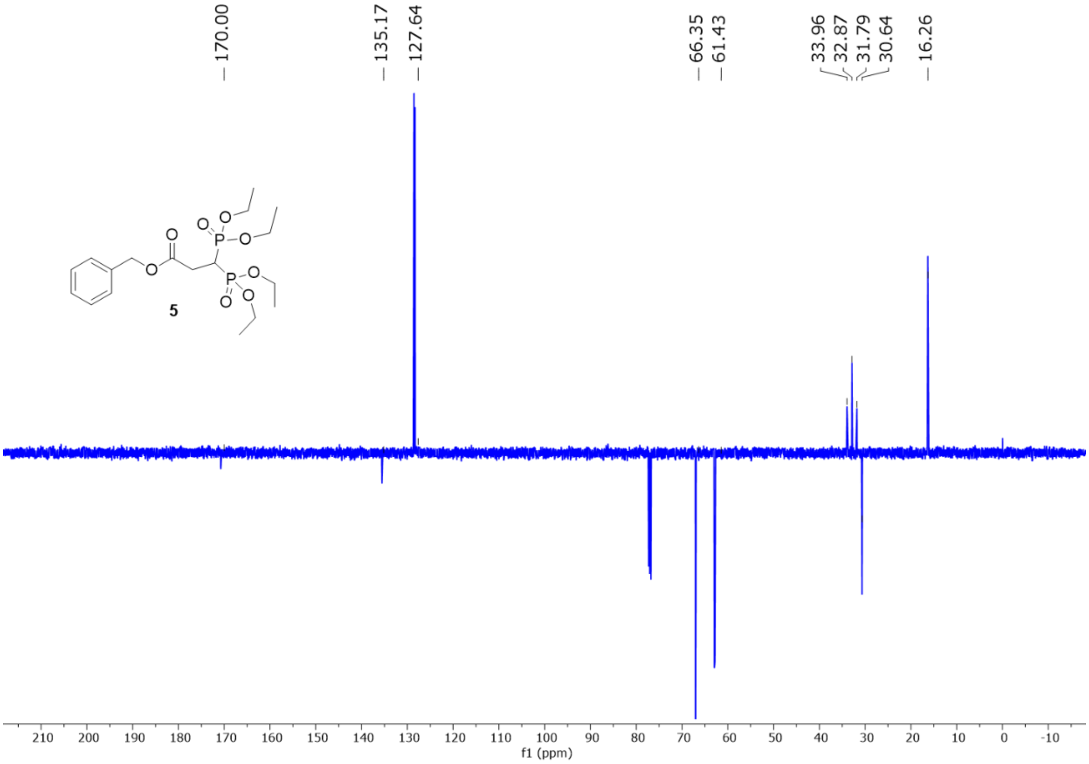

Fig. 10 Fig. 6 DEPT ^{13}C NMR spectra of bisphosphonate **5**

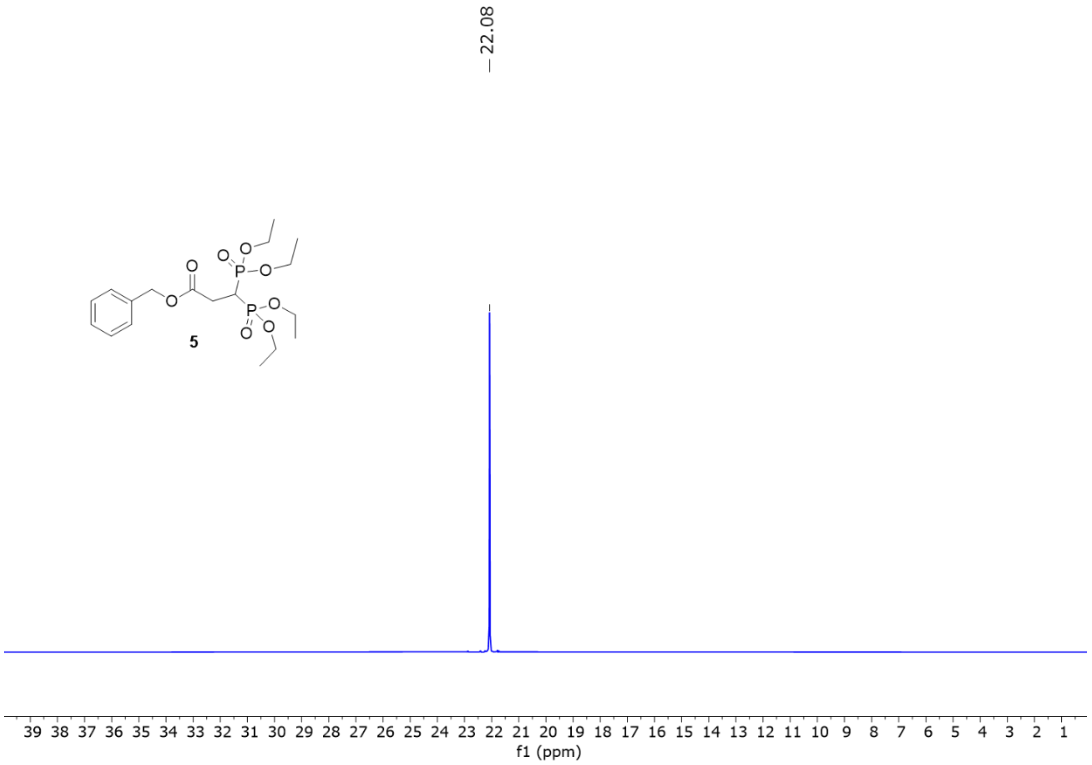

Fig. 11 ^{31}P NMR spectra of bisphosphonate **5**

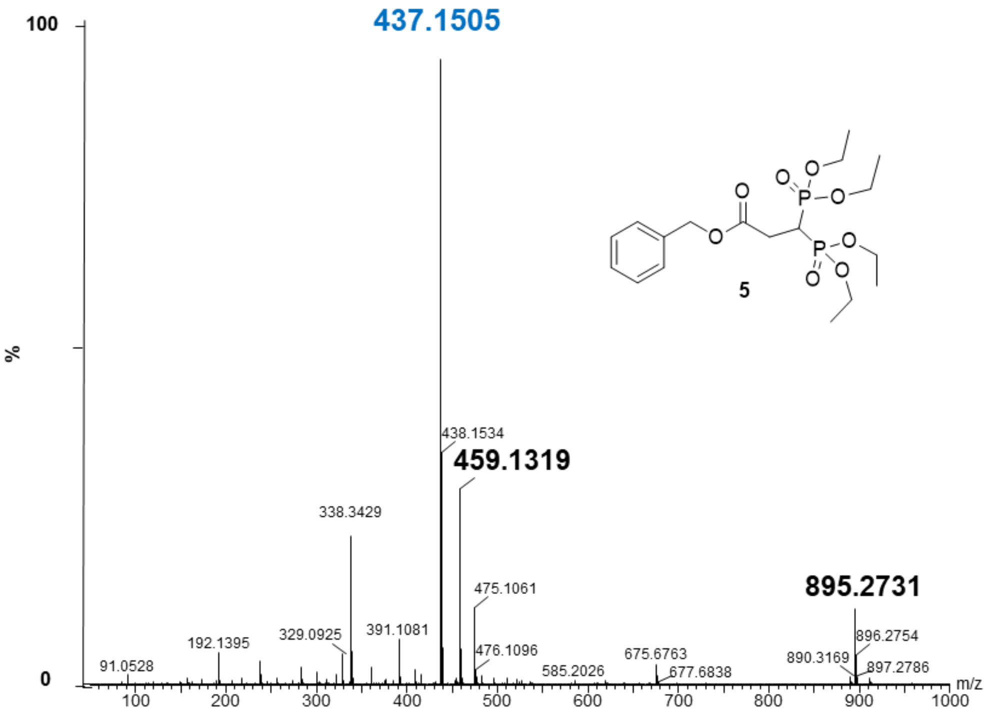

Fig. 12 HRMS spectra of bisphosphonate **5**

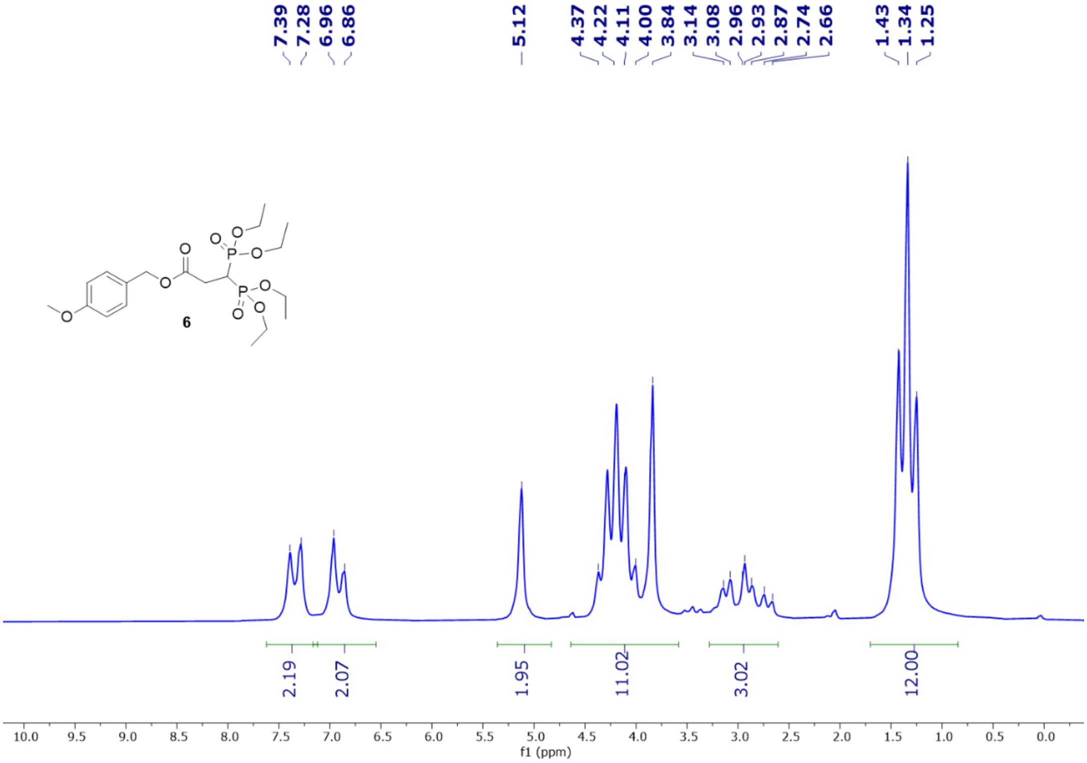

Fig. 13 ^{1}H NMR spectra of bisphosphonate **6**

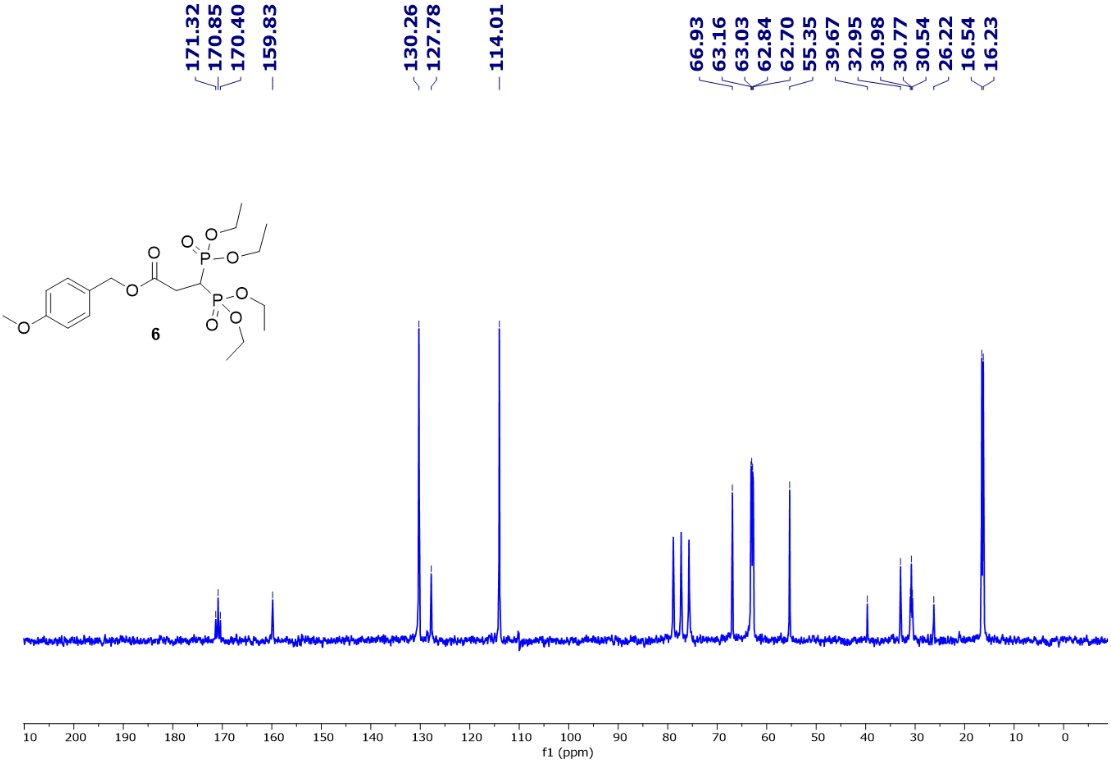

Fig. 14 ^{13}C NMR spectra of bisphosphonate **6**

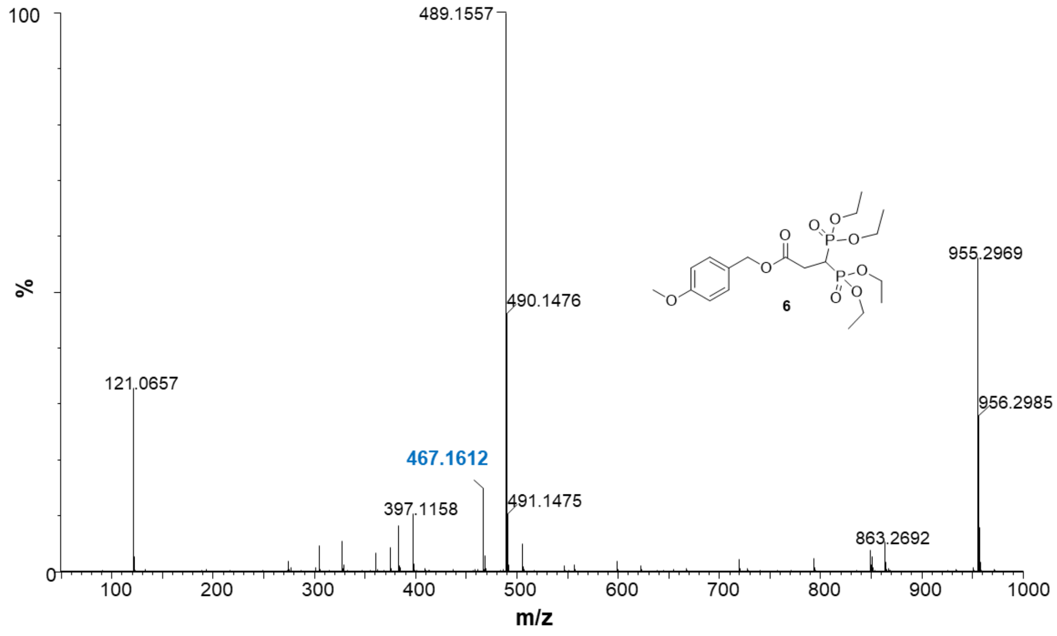

Fig. 15 HRMS spectra of bisphosphonate **6**
